# Identification of Exosomal MicroRNA Signature by Liquid Biopsy in Hereditary Hemorrhagic Telangiectasia Patients

**DOI:** 10.3390/ijms22179450

**Published:** 2021-08-31

**Authors:** Ana Pozo-Agundo, Nerea Villaescusa, Jordi Martorell-Marugán, Olga Soriano, Socorro Leyva, Ana Belén Jódar-Reyes, Luisa María Botella, Pedro Carmona-Sáez, Francisco Javier Blanco

**Affiliations:** 1 Department of Biochemistry and Molecular Biology (III) and Inmunology, School of Medicine, University of Granada, 18016 Granada, Spain; ana222pozo2@hotmail.com (A.P.-A.); n9villaescusa@gmail.com (N.V.); olgasoriano@correo.ugr.es (O.S.); 2 Centre for Biomedical Research, Biopathology and Regenerative Medicine Institute (IBIMER), University of Granada, 18016 Granada, Spain; 3 Bioinformatics Unit, Pfizer, Andalusian Government Centre of Genomics and Oncological Research (GENYO), University of Granada, 18016 Granada, Spain; jordi.martorell@genyo.es (J.M.-M.); pcarmona@ugr.es (P.C.-S.); 4 Department of Statistics and OR, University of Granada, 18016 Granada, Spain; 5 San Cecilio Clinic Universitary Hospital, 18016 Granada, Spain; socorro.leyva@gmail.com; 6 Biocolloid and Fluid Physics Group, Excellence Research Unit Modeling Nature (MNat), Department of Applied Physics, School of Sciences, University of Granada, 18071 Granada, Spain; ajodar@ugr.es; 7 Department of Molecular Biomedicine, Centro de Investigaciones Biológicas (CSIC), 28040 Madrid, Spain; cibluisa@cib.csic.es; 8 Centro de Investigación Biomédica en Red, CIBERER, Instituto de Salud Carlos III, 28040 Madrid, Spain

**Keywords:** hereditary hemorrhagic telangiectasia, liquid biopsy, exosomes, miRNAs, arteriovenous malformations

## Abstract

Hereditary hemorrhagic telangiectasia (HHT) is a rare autosomal dominant vascular dysplasia characterized by epistaxis, mucocutaneous telangiectases, and arteriovenous malformations (AVM) in the visceral organs. The diagnosis of HHT is based on clinical Curaçao criteria, which show limited sensitivity in children and young patients. Here, we carried out a liquid biopsy by which we isolated total RNA from plasma exosome samples. A cohort of 15 HHT type 1 patients, 15 HHT type 2 patients, and 10 healthy relatives were analyzed. Upon gene expression data processing and normalization, a statistical analysis was performed to explore similarities in microRNA expression patterns among samples and detect differentially expressed microRNAs between HHT samples and the control group. We found a disease-associated molecular fingerprint of 35 miRNAs over-represented in HHT vs. controls, with eight being specific for HHT1 and 11 for HHT2; we also found 30 under-represented, including nine distinct for HHT1 and nine for HHT2. The analysis of the receiver operating characteristic (ROC) curves showed that eight miRNAs had good (AUC > 75%) or excellent (AUC > 90%) diagnosis value for HHT and even for type HHT1 and HHT2. In addition, we identified the cellular origin of these miRNAs among the cell types involved in the vascular malformations. Interestingly, we found that only some of them were incorporated into exosomes, which suggests a key functional role of these exosomal miRNAs in the pathophysiology of HHT.

## 1. Introduction

Hereditary hemorrhagic telangiectasia (HHT) or Rendu–Osler–Weber syndrome is a vascular dysplasia with autosomal dominant traits characterized by recurrent and spontaneous nose bleeds (epistaxis), mucocutaneous telangiectases, and arteriovenous malformations (AVM) in the internal organs, including the lungs, liver, and brain [1]. Mutations in the encoding gene for the transforming growth factor (TGF)-β receptors endoglin (ENG) and Activin A receptor type II-like 1, ALK1 (ACVRL1), lead to HHT type 1 (MIM #187300) and type 2 (MIM #600376), respectively [2]. Both types account for the majority of the genetically diagnosed HHT population (85% approximately), although recent reports point to mutations in other components of the TGF-β signaling pathway, such as SMAD4 (MIM #175050) and BMP9 (GDF2) (MIM #615506), in a reduced HHT population [1]. In any case, HHT is a rare disease with an estimated prevalence around 1/8000. The disease diagnosis is based on the clinical symptoms gathered in the Curaçao criteria, which include frequent and recurrent epistaxis, multiple telangiectases, visceral AVM, and a first-degree relative with HHT according to these criteria. However, HHT patients may appear to have no symptoms until their epistaxis is strong, around the third or fourth decade of life. They may even have AVM in the lungs or brain before the onset of epistaxis and telangiectases, which raises the hypothesis that not only genetics but a second event must occur to trigger the disease [3]. This situation usually causes a delay in diagnosis of many years, which prompts the need for early molecular diagnosis.

Liquid biopsy is a non-invasive technology for the detection of molecular biomarkers without the necessity of expensive or invasive procedures [4]. Circulating extracellular vesicles (EVs) can be isolated from blood and other biological fluids, and the analysis of the cargo may provide key information about a specific condition or disease. In this sense, plasma exosomes are crucial EVs for this purpose [5]. Exosomes are small vesicles (<200 nm in diameter) with a low refractive index derived from multivesicular bodies continuously secreted by a vast number of cell types to the extracellular environment, and they represent a novel vehicle for cell-to-cell communication and epigenetic regulation. This is mainly due to their inherent property to convey bioactive molecules, including small noncoding RNA such as microRNAs, among others [6].

MicroRNAs (miRNAs) are a large family of small (22–24 nucleotides long), non-coding RNAs that play a critical role in a variety of physiological and pathological processes. In the cell, miRNAs mediate post-transcriptional gene silencing by binding to the 3′-untranslated region (UTR) of target mRNAs, suppressing in this way their translation into protein [6]. Interestingly, it has been demonstrated that the repertoire of miRNAs is not randomly incorporated into exosomes [7]. Hence, the characterization of the exosome-transported miRNAs is a pioneer area of investigation with a high impact on clinical diagnosis and prognosis, as well as on the response to therapy. In this sense, small RNA sequencing by RNA-seq has emerged as a powerful tool in transcriptomics, gene expression profiling, and biomarker discovery [5]. Sequencing exosome-transported miRNAs from liquid biopsy provides exciting possibilities for molecular diagnosis and might help to establish disease-specific biomarker signatures.

In the present work, we have analyzed for the first time the miRNA cargo in plasma exosomes from HHT patients, obtaining the molecular signature of differentially expressed miRNA in HHT. We have selected a set of eight miRNAs with high diagnostic value for HHT, which allow us to discriminate even between type 1 and 2. Moreover, the bioinformatics analysis points out the biological functions affected in HHT. Finally, we have identified the producer and receptor cell type for the exosomal miRNAs, suggesting a hitherto unknown functional role for the cell-to-cell communication in the physiopathology of HHT.

## 2. Results

### 2.1. The Exosome Isolation Shows Similarity among Samples

**Figure 1:**
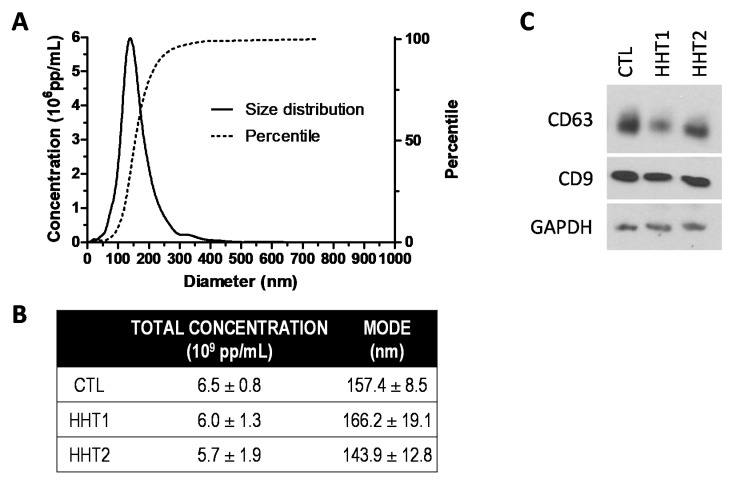
Plasma exosome characterization. (**A**) A representative graph from NTA analysis for size distribution and concentration determination of plasma exosomes is shown. (**B**) The total concentration and diameter mode of exosomes were calculated for CTL, HHT1, and HHT2 groups (mean ± standard deviation). (**C**) Classical exosome markers were detected by Western blot.

The characterization of plasma exosomes derived from different experimental groups was analyzed by Nanoparticle Tracking Analysis (NTA). The total concentration of particles expressed as 10^9^ pp/mL from 500 μL plasma was 6.5 ± 0.8 (CTL), 6.0 ± 1.3 (HHT1), and 5.7 ± 1.9 (HHT2). The diameter of the particles, expressed as the mean of the mode values in nm, was 157.4 ± 8.5 (CTL), 166.2 ± 19.1 (HHT1), and 143.9 ± 12.8. These results are in line with the features described for plasma exosomes elsewhere. No significant differences were obtained either for concentration or size among groups (Figure 1A,B). Plasma exosomes were positive for the expression of tetraspanins CD63 and CD9 (Figure 1C). We observed slight differences in the expression levels of these exosome markers in some HHT samples. However, it was not a reliable and reproducible result for all samples within a specific HHT group, so that we cannot conclude any correlation associated with the disease.

### 2.2. MiRNAs Signatures Differentiate HHT Subtypes and Healthy Samples

**Figure 2:**
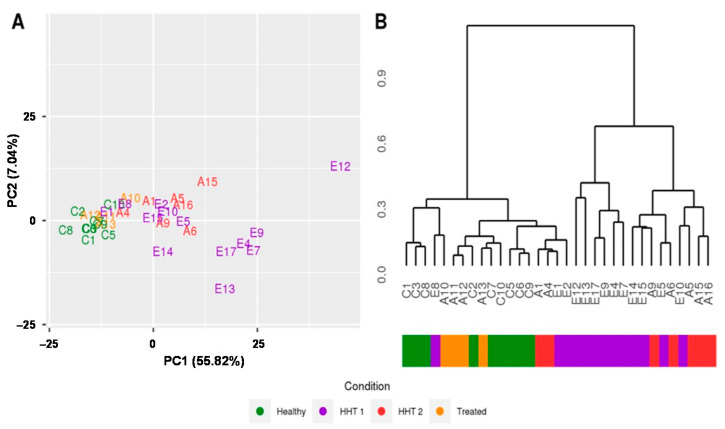
Comparison of HHT and healthy samples from miRNA gene expression signatures. (**A**) Principal component analysis and (**B**) hierarchical clustering analysis show that global gene expression signatures are grouped into two main clusters, one containing healthy (green) and treated patients (yellow) and the other containing most of the disease samples from HHT1 (purple) and HHT2 (red) subtypes.

Gene expression data (RNA-Seq data) were processed and normalized as described in the Methods section, and 179 miRNAs expressed in at least 75% of samples were used for characterizing gene expression signatures in healthy and HHT samples. In order to explore the similarities in samples based on the global microRNA expression profiles, we applied principal components analysis (PCA) and hierarchical clustering. PCA showed a separation of healthy and HTT samples, with the first component with an explained variance of 55% being the most relevant one that clearly distinguished both groups (Figure 2A). In addition, the clustering analysis revealed that a set of samples from the HHT1 subtype were placed in a separate cluster, revealing that the miRNA expression profiles provided a clear three-cluster structure that matched with the disease phenotypes (Figure 2B). Furthermore, we also observed that some HHT samples were clustered within the healthy group. Interestingly, these patients had been previously submitted to a reparatory surgery to eliminate telangiectases from nasal mucosa by sclerotherapy. Therefore, this suggests that vascular anomalies are the source of plasma exosomes that bear the specific miRNAs in HHT and the role of the miRNA signature as an active disease biomarker.

### 2.3. MiRNAs Differentially Expressed among HHT Patients and Control Samples

**Figure 3:**
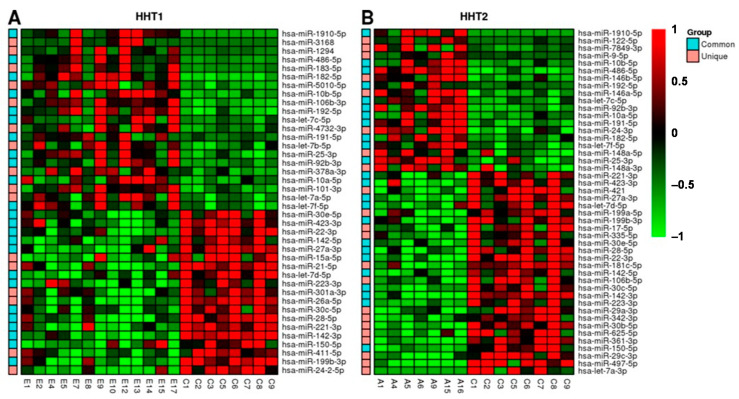
Heatmap showing the most differentially expressed miRNAs (adjusted *p*-value < 0.05) among (**A**) HHT1 subtype and healthy samples and (**B**) HHT2 subtype and healthy samples. MicroRNAs are in rows and individual samples are shown in columns, and their names refer to the corresponding experimental group: C, control; E, HHT1; and A, HHT2. Red and green colors represent upregulation and downregulation, respectively. The single left-hand column in each panel shows those miRNAs that were found significant in the comparison of all HHT samples versus healthy, marked as common (cyan), while those that were found significant in each subtype are marked as unique (pink).

In order to define the set of miRNAs differentially expressed between disease and healthy samples, we performed pairwise comparisons among non-treated HHT samples and healthy individuals as well as HHT subtypes and healthy samples. The rationale of these pairwise comparisons was not only to detect those miRNAs that were deregulated in HHT samples with respect to healthy ones, but also those miRNAs with subtype-specific deregulation. Using the DESeq2 R package, we obtained 40 miRNAs that were differentially expressed between HHT1 subtype patients and healthy samples (Figure 3A). On the other hand, we identified 46 miRNAs differentially expressed between HHT2 subtype patients and healthy samples (Figure 3B). Furthermore, we compared all HHT samples with healthy ones, finding 44 miRNAs differentially expressed between both types of HHT and healthy samples. Most of these miRNAs were also found in the individual subtype comparisons (adjusted *p*-value < 0.05). The complete list of differentially expressed miRNAs is available in the additional material (Supplementary Table S1), in which some miRNAs have been previously described to be associated with HHT, such as miR-27a-3p [8], miR-28-5p, or miR-361-3p [9]. Furthermore, we identified several miRNAs whose expression in plasma exosomes was not altered in HHT with respect to the healthy group. This provided us with the chance to use them as an internal control for the further validation of these results by real-time PCR.

To discover the main biological processes associated with the miRNAs differentially expressed in HHT, we analyzed the enrichment of biological pathways in the set of target genes. To this end, we retrieved target genes that were supported by strong experimental evidence from miRTarBase. The most significant pathways are shown in Table 1 (see Supplementary Table S2 for complete list). Target genes were enriched in pathways such as angiogenesis, the p53 pathway, PDGF signaling, or the TGF-β signaling pathway, among others.

### 2.4. HHT-Associated miRNAs Have Diagnostic Value

Next, we wanted to validate by real-time PCR some representative miRNAs from the RNA-seq results. To this end, we employed the TaqMan^TM^ Advanced assay due to its high sensitivity and specific quantification of mature miRNA, since the miRNA concentration in exosomes is very low. We chose those with at least a two-fold change in absolute value terms, i.e., up- and downregulated miRNAs (|log_2_FoldChange| > 1), and an adjusted *p*-value < 0.05. Unfortunately, an external factor that conditioned our miRNA selection was the commercial availability of these assays for the real-time PCR experiments. Then, we selected a different cohort of HHT patients and healthy donors (7 donors per group), isolated exosomes from plasma, and extracted total RNA. We assayed miR-9-5p, miR-29c-3p, miR-142-3p, miR-150-5p, miR-183-5p, miR-486-5p, miR-654-3p, and miR-106b-3p. In addition, miR-103a was used as the internal control (housekeeping target) since it showed almost a null fold change with the lowest FDR. In parallel, we measured cel-miR-39-3p as the spike-in control. Hereafter, the miRNA strand assayed by real time PCR is omitted to simplify their names.

**Figure 4:**
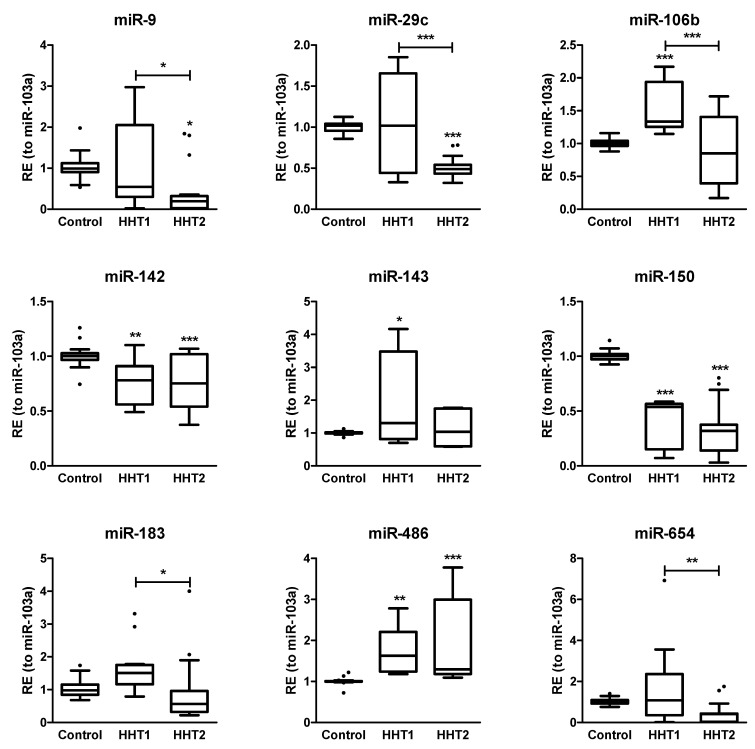
Validation of the miRNA signature of HHT. The relative expression (RE) of 9 miRNAs was assayed by real-time PCR in a cohort of 7 donors per group. The comparison with the control group was carried out by the analysis of variance test (ANOVA) with the Bonferroni correction (* *p* < 0.05; ** *p* < 0.01; *** *p* < 0.005). Tukey’s method was applied to identify outliers (black dots) in the box-plots.

We corroborated the dysregulated expression level obtained by RNA-seq of some miRNAs in the HHT versus healthy group, while the expression of some others was affected only in a subtype of HHT (Figure 4). Thus, we verified that the expression of miR-142, miR-150, and miR-486 in plasma exosomes was altered in both HHT1 and HHT2 with respect to the control group, which suggests that these miRNAs might be general biomarkers for HHT. Moreover, we confirmed a statistically significant overrepresentation of miR-106b and miR-143 in plasma exosomes from HHT1 patients versus the control group, and that for miR-183 and miR-654 but in HHT1 versus HHT2. This fact confers to these miRNAs a putative discriminatory role for diagnosis between HHT subtypes. In this context, we corroborated a decrease in plasma exosomal miR-9 and miR-29c specifically in HHT2 with respect to the control and HHT1 groups, which reinforces the ability of our data to classify the patients as HHT1 and HHT2.

**Figure 5:**
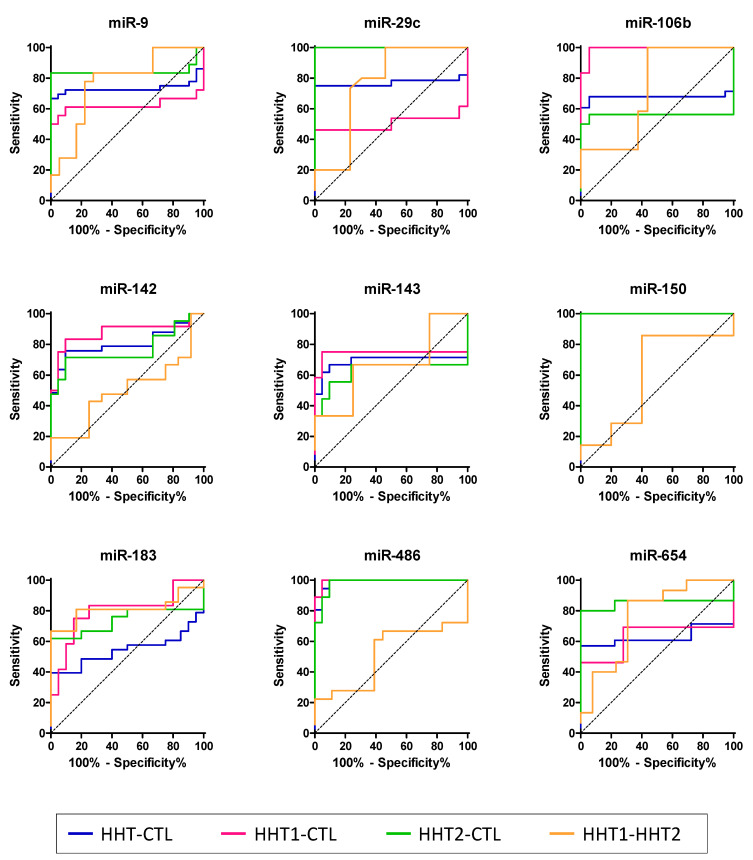
Analysis of the area under the ROC curves (AUC). ROC curves were calculated for the 95% confidence interval. Of note, the color of some ROC curves—for example, in miR-150—is missing due to overlapping with others (see Table 2 for AUC values).

Nonetheless, we wanted to determine the strength of these miRNAs as biomarkers of HHT. Thus, the receiver operating characteristic (ROC) curve and area under the ROC curve (AUC) analysis was used to evaluate their predictive ability. We carried out all comparisons with respect to the control group (Figure 5). AUC values demonstrated that most of the miRNAs analyzed from the selected profile are good (AUC > 75%) and excellent (AUC > 90%) biomarkers for HHT, even allowing us to distinguish between HHT1 and HHT2 (Table 2). However, we found that although miR-143 is increased in plasma exosomes from HHT1 patients, it cannot be considered a biomarker since its AUC is lower than 75%.

### 2.5. Screening the Cellular Source of Exosomal miRNAs

Since we observed that the exosomal miRNA expression profile associated with HHT was restored to that of the healthy group after a reparatory surgery on the AVM-affected tissue in some patients, we decided to identify the cells responsible for this HHT-associated signature. Therefore, we cultured endothelial (PAEC) and smooth muscle cells (PASMC) from the pulmonary artery. In addition, we included in the analysis macrophages subjected to either classical (M1) or alternative (M2) activation, since they are part of the inflammatory cell infiltrate present in pulmonary AVM and in many other tissues, such as the skin and brain. Thus, we contrasted in parallel the miRNA expression in their cytoplasm and exosomes isolated from conditioned supernatants (Table 3).

First, we corroborated that our internal reference miR-103a was expressed by all cell types and transported in their exosomes. The RT-qPCR results demonstrated clear differences regarding the other miRNAs depending on the producer cell type not only in the cytoplasm expression but also in the exosome cargo. Thus, miR-29c and miR-106b are expressed by all cell types, as analyzed in their cytoplasm and in their exosome cargo. By contrast, miR-150 was only detected in the cytoplasm and exosomes from PAECs. Interestingly, miR-143 is expressed by all cell types but is only transported in exosomes from PASMCs. MiR-142, miR-183, and miR-9 are expressed and included in exosomes by M1- and M2-polarized macrophages. In addition, both macrophage types and PAECs express miR-486, but only PAECs incorporate it into exosomes. In the case of miR-654, it is synthesized by both vascular cell types, PAECs and PASMCs, but is only detected in PASMC exosomes (Table 3).

## 3. Discussion

A common feature among rare diseases, and HHT in particular, is that their diagnosis is a time-consuming process from the onset of the very first symptoms. In this sense, liquid biopsy could be a quick and valuable method for a precise diagnosis when an individual is suspected of having HHT. Therefore, molecular biomarkers that allow an early HHT diagnosis would have a significant impact on clinical practice.

Here, we have identified a specific miRNA signature by liquid biopsy in plasma exosomes from HHT patients, which allows us not only to identify patients but also to discriminate between those of type 1 and 2. To date, only a few miRNAs in plasma have been reported to be associated with HHT, including miR-205 and miR-27a [8], and miR-210 [10]. However, these miRNAs were not identified as part of the exosome cargo, and the method used for their identification was based on commercial miRNA cards. In the present work, we have carried out RNA-seq screening of exosomes isolated from plasma in order to identify the whole RNA content. In this way, we have avoided obtaining biased results that would depend on the probes immobilized in the arrays (cards) previously reported elsewhere.

We found that miRNA expression profiles clearly differentiate HHT patients from healthy individuals, revealing these miRNA signatures as potential biomarkers for this disease. Moreover, those samples obtained after reparatory surgery of AVM (treated samples) showed greater similarity to control samples than non-treated patients, which reinforces the idea that miRNA expression is altered in the pathological state and the elimination of AVM reverts the pathological gene expression signature. The differential expression analysis between exosomes from HHT patients and healthy donors (Supplementary Table S1) yielded 65 miRNAs whose expression was significantly different. Upon validation of a miRNA set including those with the most significant fold change rates, we next calculated their diagnostic value by studying ROC curves. Thus, 8 out of 9 miRNAs are strong candidates for HHT biomarkers (AUC > 75%). Indeed, we found that, although miR-143 is differentially increased in HHT1 samples, it cannot be considered a biomarker as it showed an AUC < 75% for any comparison group. However, this does not exclude the possibility that miR-143 plays a role in HHT. In fact, miR-143 has been reported to be expressed by vascular smooth muscle cells, transported in exosomes and acting on the nearby endothelial cells. This has been described in the plexiform lesions of patients with pulmonary arterial hypertension, a vascular disease closely related to HHT [11].

We are aware that the sample size for the validation set is limited and a large cohort would be necessary in most of the cases, as the AUC is lower than 0.9. Nevertheless, the trend found for the most significant ones is indicative of their potential role as biomarkers, but further validations will be required to effectively establish these miRNAs as good diagnostic markers for clinical practice.

On the other hand, miR-142 and miR-150 emerged as excellent biomarkers with diagnostic value for HHT. Besides their roles as HHT biomarkers, here, we demonstrate that miR-142 and miR-150 are synthesized and released in exosomes by macrophages and endothelial cells, respectively. Thus, we can hypothesize their biological functions in AVM, as it was reported by different groups that both miRNAs act as tumor suppressors in several cancer types, increasing the tumor cells’ progression [12,13,14]. Therefore, since these miRNAs are transported in low levels in exosomes from HHT patients compared to the healthy group, it would suggest a higher proliferation rate for vascular cells that would contribute to the AVM formation and/or progression in HHT. In particular, miR-150 is decreased in pulmonary hypertension patients, and pharmacological restoration in animal models has beneficial effects by protecting against hypoxia-induced pulmonary vascular remodeling, fibrosis, and abnormal proliferation of PASMCs and PAECs [15].

Furthermore, we found that miR-486 is overrepresented in HHT exosomes and is a superb biomarker with diagnostic value for HHT. Here, we have found that miR-486 is synthesized by macrophages and endothelial cells, but only the latter load it into exosomes. This suggests that smooth muscle cells could be the main receptor cells for miR-486 within the AVM context. Recent papers point out a role for miR-486 in the cardiovascular system, including angiogenesis and myocardial infarction [16,17], or even as a biomarker of cardiac pathologies such as bicuspid aortic disease [18]. Thus, we hypothesize that the increase in miR-486 in exosomes might alter the vascular remodeling, contributing to the development of AVM in HHT.

In addition, we have reported other exosome-transported miRNAs as HHT biomarkers that even help to discriminate between the type 1 and 2 forms of the disease. This is the case for miR-106b, miR-654, and miR-183, which are specifically augmented in HHT1. In the AVM cellular context, miR-106b is synthetized by all three cell types, miR-654 is exclusively released in exosomes by smooth muscle cells, and exosomal miR-183 has a macrophage origin. Unfortunately, little is known about the role of these three miRNAs in the vasculature, but it has been reported previously that they are related to atherosclerosis and angiogenesis promotion [19,20,21]. For HHT2, miR-9 and miR-29c are underrepresented in exosomes from these patients. While the former is a Myc-activated miRNA, expressed and released by macrophages, that promotes angiogenesis [22,23], the latter seems to act as a tumor suppressor in diverse cancer types [24,25]. Thus, low levels of both miRNAs would contribute to the physiopathology of AVM, controlling vascular cell proliferation. Our current investigations are focused on the intercellular communication mediated by these exosomal miRNAs associated with HHT and the role that they may carry out in the development of AVM, as well as their putative role as therapeutic targets.

## 4. Materials and Methods

### 4.1. Volunteers and Blood Samples, Exosome Isolation, and miRNA Extraction and Sequencing

Informed consent was obtained from all volunteers participating in the study, in accordance with the regional Ethics Committee for Investigation (CEI) of Granada, Spain. Genetically tested HHT patients, also positive for the Curaçao criteria, were assigned to the HHT1 (*n* = 15, numbered as E#) and HHT2 (*n* = 15, numbered as A#) groups depending on whether their mutation was in the ENG or ACVRL1 gene, respectively. Non-HHT donors participated as the control group (CTL; *n* = 10, numbered as C#). Volunteers’ age ranged from 25 to 65 years old. No children were included in the study.

Venous blood samples (5 mL) were collected in sodium citrate tubes and processed within the following 24 h. Blood cells were removed by centrifugation at 1500 g for 10 min at 4 °C. Next, platelets were depleted from the supernatant by centrifugation at 2000 g for 15 min. Resulting plasma was 500 μL-aliquoted and stored at −20 °C until exosome isolation.

### 4.2. Exosome Isolation and miRNA Extraction

Plasma aliquots and conditioned culture media were pre-cleared at 10,000 g for 20 min at 4 °C. Then, supernatants were analyzed with the Total Exosome Isolation Kit from plasma and from cell culture, respectively (Thermo Fisher, Carlsbad, CA, USA), following the manufacturer’s instructions. These protocols are based on a proteinase K digestion and successive centrifugation steps in the presence of a precipitating reagent, until the final resuspension of the exosomes in PBS buffer. Exosomes were processed and analyzed immediately. Alternatively, exosomes were stored at 4 °C for 24 h maximum or −20 °C for extended storage to avoid aggregate formation.

The hydrodynamic size distribution of the exosomes (particle concentration vs. diameter) was measured by Nanoparticle Tracking Analysis (NTA) in a NanoSight LM10-HS(GB) FT14 (NanoSight LTD, Amesbury, United Kingdom) equipped with a high-sensitivity EMCCD camera and a sample chamber with a 405 nm laser. The video images of the particles, in Brownian motion, were captured and analyzed by the NTA 2.3 image analysis software. All samples were measured at least in triplicate with manual shutter, gain, brightness, and threshold adjustments at 25 °C. Then, total RNA was extracted using the Total Exosome RNA and Protein Isolation Kit (Thermo Fisher, Carlsbad, CA, USA), according to the manufacturer’s instructions. The quality of the isolated RNA was determined in a 2100 Bioanalyzer in the pg/μL sensitivity range (Agilent, Santa Clara, CA, USA). In parallel, total RNA from cells was obtained using the miRNeasy kit (Qiagen, Hilden, Germany), following the manufacturer’s instructions. In addition, protein analysis by Western blot for CD63 (Ts63; Thermo Fisher, Carlsbad, CA, USA) and CD9 (Ts9; Thermo Fisher, Carlsbad, CA, USA) was carried out to test the purity of the exosome isolation.

### 4.3. Small RNA Sequencing

Quality control was performed using the Agilent 2100 Bioanalyzer with the Eukaryote Total RNA Pico Kit and High Sensitivity DNA Assay (Agilent Technologies, Santa Clara, CA, USA) to assess total RNA quantity and quality. Libraries were prepared using the TruSeq Stranded mRNA Library Preparation Kit (Illumina Inc., San Diego, CA, USA), and RNA sequencing was carried out on an Illumina NextSeq 500 System.

### 4.4. Retrotranscription and Quantitative PCR (RT-qPCR)

Exosomal miRNAs from 500 μL of plasma were spiked-in with the irrelevant cel-miR-39-3p at 200 pM as an exogenous control and then retrotranscribed to cDNA using the TaqMan Advanced miRNA cDNA Synthesis Kit (Thermo Fisher, Carlsbad, CA, USA). Briefly, it uses 3’ poly-A tailing and 5’ ligation of an adaptor sequence to extend the mature miRNAs present in the sample on each end prior to reverse transcription. Universal RT primers recognize the universal sequences present on both the 5’ and 3’ extended ends of the mature miRNAs. Thus, all mature miRNAs in the sample were reverse-transcribed to cDNA. Next, the cDNA was amplified using the Universal miR-Amp Primers and miR-Amp Master Mix to uniformly increase the amount of cDNA for each target, maintaining the relative differential expression levels and improving the detection of low-expressing miRNA targets. Then, a real-time PCR with TaqMan Advanced miRNA Assays was carried out for each miRNA in a QuantStudio3 system. Data were normalized to miR-103a-3p (invariable internal control) values for miRNA expression using the 2^−ΔΔCt^ method. Results were expressed as fold change relative to the control. The qPCRs were run in triplicate and results are presented as the mean ± standard error of samples.

### 4.5. Data Processing and Statistical Analysis

Entire RNA-Seq data analysis was performed using the QuickMIRSeq suite [26]. Briefly, raw sequences were trimmed in order to remove adapter sequences introduced during the library preparation. Reads shorter than 15 nucleotides (nt) and larger than 28 nt after trimming were discarded to select those sequences more likely to map against miRNAs. Reads were aligned to human reference genome GRCh38. Only miRNAs with 5 or more counts in at least 25% of samples were used in the differential expression analysis [27] and raw counts were normalized with trimmed mean of M values (TMM) method [28]. Finally, we used the DESeq2 package [29] to detect miRNAs that were differentially expressed among the different classes. PCA was performed with the R package NOISeq [30] using miRNAs with 5 or more counts in at least 75% of samples, and hierarchical clustering analysis was performed applying Ward’s method [31] and Pearson’s correlation distance to these filtered reads.

A validation cohort for the miRNA signature of HHT was selected for each experimental group (*n* = 7), different to the cohort taken for RNA-seq analysis. RT-qPCR experiments were carried out as described previously and results were represented in box-plots, where whiskers were calculated using Tukey’s method with the GraphPad Prism software.

The diagnostic value of miRNA signature was determined by the receiver operating characteristic (ROC) curve analysis. The areas under the ROC curves (AUC) were calculated for the 95% confidence interval, taking *p* < 0.05 as statistically significant. Sensitivity and specificity were calculated for each miRNA.

Sample size estimation using AUC corresponding to a 5% alpha error, 10% beta error, AUC of 0.98, and ratio of sample size in negative and positive groups of 1 yielded a *n* = 8.

We used R, GraphPad Prism software, and MedCalc software for data.

### 4.6. Analysis of Functional Terms

To characterize the main biological functions associated with the list of significant miRNAs, we first retrieved target genes associated with each microRNA supported by strong experimental evidence (reporter assay or Western blot) using information from miRTarBase [32] and miRWalk [33]. Overrepresentation of functional annotations in target genes were then evaluated by means of a binomial test implemented in Panther [34]. Briefly, we assumed the ‘null’ hypothesis as the genes in the input and reference list were sampled from the same population.

The probability *P(A)* of observing a gene from a category *A* in the reference list is given by:

P(A)=n/N

where *n* is the number of genes belonging to the analyzed category and *N* the genes in the reference list. Then, the probability of observing *k* genes belonging to category *A* in the input list of size *K* is calculated for over- and underrepresentation as:

$$p\text{-}val_{over\text{-}representation}=\sum_{i=k}^{K}\left(\begin{array}{c} K\\ i \end{array}\right){\left(A\right)}^{i}{\left(1-P(A)\right)}^{K-i}$$

$$p\text{-}val_{under\text{-}representation}=\sum_{i=0}^{k}\left(\begin{array}{c} K\\ i \end{array}\right){\left(A\right)}^{i}{\left(1-P(A)\right)}^{K-i}$$

Those pathways with FDR-adjusted *p*-value less than 0.05 were selected as significant.

### 4.7. Cell Culture

Primary cultures of human pulmonary artery endothelial cells (PAECs) and smooth muscle cells (PASMCs) were commercially acquired (Promocell, Heidelberg, Germany). PAECs and PASMCs were cultured at low passages in Endothelial Cell Growth Medium 2 Kit and Smooth Muscle Cell Growth Medium 2 Kit (Promocell, Heidelberg, Germany), respectively. PAECs were seeded onto 0.2% gelatin-coated plates.

For macrophage polarization, we followed the protocol described by Genin et al. [35]. Briefly, human monocytic THP-1 cells were maintained in RMPI 1640 (Gibco, Life Technologies, Paisley, UK) supplemented with 10% of heat-inactivated fetal calf serum (FCS), GlutaMAX (Gibco, Life Technologies, Paisley, UK), 10 mM Hepes, 1 mM sodium pyruvate, and 50 pM β-mercaptoethanol. First, THP-1 monocytes were differentiated into macrophages by 24-h incubation with 10 ng/mL phorbol 12-myristate 13-acetate (Sigma-Aldrich, Saint Louis, MO, USA) followed by 24-h incubation in complete RPMI medium. Then, cultures were polarized in either M1 or M2 macrophages by incubation with 1 μg/mL LPS (Sigma-Aldrich, Saint Louis, MO, USA) and 20 ng/mL IFN-γ (PeproTech, Cranbury, NJ, USA), or 20 ng/mL IL-4 (PeproTech, Cranbury, NJ, USA) and 20 ng/mL IL-13 (PeproTech, Cranbury, NJ, USA), respectively, for 48 h.

All cells were maintained in an incubator at 37 °C with humidified atmosphere and 5% CO2. For exosome isolation, the FCS from the culture media was replaced with exosome-depleted FCS (Gibco, Life Technologies, Paisley, United Kingdom) prior to the recovery of the conditioned supernatants.

## 5. Conclusions

Here, we report for the first time the specific signature of differentially expressed miRNAs transported in plasma exosomes associated with HHT. Furthermore, the majority of them have strong diagnostic value and allow us to discriminate between HHT1 and HHT2. Our data indicate that the cellular components of AVM determine this exosomal miRNA signature. The role of these exosome-transported miRNAs is presently under investigation in our group and will contribute to unraveling the molecular mechanisms underlying the physiopathology of HHT.

## 6. Patents

FJB, PCS, and LMB are scientific authors of a patent with application number P201930342 in the Spanish Patent Office and International application No. PCT/ES2020/070242 owned by Universidad de Granada (UGR), Fundación Progreso y Salud, and Consejo Superior de Investigaciones Científicas (CSIC) on the use of exosomal miRNAs for the diagnosis of HHT.

## Figures and Tables

**Table 1:** Enrichment analysis results. Table shows top 20 pathways enriched (FDR < 0.05) in the list of target genes. Fold enrichment (FE) values greater than 1 indicate that the pathway annotation is overrepresented in the experiment and less than 1 indicates that it is underrepresented.

Panther Pathways ^1^	Genes in Reference ^2^	Genes in List ^3^	FE ^4^	FDR ^5^
Gonadotropin-releasing hormone receptor pathway (P06664)	230	64	6.41	2.13 × 10^−26^
CCKR signaling map (P06959)	174	54	7.15	3.27 × 10^−24^
Apoptosis signaling pathway (P00006)	118	45	8.79	3.39 × 10^−23^
p53 pathway feedback loops 2 (P04398)	51	30	13.56	1.42 × 10^−19^
Angiogenesis (P00005)	173	47	6.26	2.69 × 10^−19^
p53 pathway (P00059)	87	33	8.74	4.09 × 10^−17^
EGF receptor signaling pathway (P00018)	134	39	6.71	5.68 × 10^−17^
Interleukin signaling pathway (P00036)	89	32	8.29	3.72 × 10^−16^
Ras pathway (P04393)	74	29	9.03	1.69 × 10^−15^
Inflammation mediated by chemokine and cytokine signaling pathway (P00031)	260	50	4.43	2.27 × 10^−15^
PDGF signaling pathway (P00047)	148	36	5.61	8.73 × 10^−14^
TGF-beta signaling pathway (P00052)	97	28	6.65	2.46 × 10^−12^

^1^ Pathway name and code in parentheses. ^2^ Number of genes in the reference that are associated with a given pathway. ^3^ Number of genes in the input list associated with a given pathway. ^4^ FE: Fold enrichment, the number in the input list divided by the expected number. ^5^ FDR: Benjamini–Hochberg corrected *p*-values.

**Table 2:** The diagnostic efficiency of the miRNA signature. The area under the ROC curve (AUC) was used to gauge and compare biomarkers’ (miRNAs) performance, classifying the biomarkers as good (yellow, 90% < AUC < 75%) and excellent (green, AUC > 90%). Sensitivity and specificity are indicated. All values are shown together with their corresponding confidence interval (95% CI).

		AUC			Sensitivity		Specificity	
		%	95% CI	*p*-Value	%	95% CI	%	95% CI
**miR-9**	**HHT-CTL**	73.3	59.3–87.4	0.0036	66.7	49.0–81.4	100.0	83.9–100.0
	**HHT1-CTL**	62.2	40.7–83.8	0.1951	61.1	35.7–82.7	90.5	69.6–98.9
	**HHT2-CTL**	84.4	68.2–100.1	0.0003	83.3	58.6–96.4	100.0	83.9–100
	**HHT1-HHT2**	76.9	60.9–92.9	0.0059	77.8	52.4–93.6	77.8	52.4–93.6
**miR-29c**	**HHT-CTL**	77.0	61.8–92.2	0.0022	75	55.1–89.3	100.0	81.5–100.0
	**HHT1-CTL**	50.4	24.4–76.5	0.9681	46.15	19.2–74.9	100.0	81.5–100.0
	**HHT2-CTL**	100.0	100.0–100.0	<0.0001	100.0	78.2–100.0	100.0	81.5–100.0
	**HHT1-HHT2**	76.7	57.4–95.9	0.0166	100.0	78.2–100.0	53.9	25.1–80.8
**miR-106b**	**HHT-CTL**	67.7	50.5–84.8	0.0452	67.9	47.6–84.1	94.4	72.7–99.9
	**HHT1-CTL**	99.1	96.7–100.0	<0.0001	100.0	73.5–100.0	94.4	72.7–99.9
	**HHT2-CTL**	55.9	31.7–80.1	0.5575	50.0	24.7–75.3	100.0	81.5–100.0
	**HHT1-HHT2**	72.4	52.9–91.9	0.0459	56.3	29.9–80.2	100.0	73.5–100.0
**miR-142**	**HHT-CTL**	80.7	68.8–92.5	0.0002	75.8	57.7–88.9	90.5	69.6–98.8
	**HHT1-CTL**	87.7	72.6–100.0	0.0004	83.3	51.6–97.9	90.5	69.6–98.8
	**HHT2-CTL**	76.6	61.1–92.1	0.0031	71.4	47.8–88.7	90.5	69.6–98.8
	**HHT1-HHT2**	50.4	30.2–70.6	0.9701	100.0	83.9–100.0	8.3	0.2–38.5
**miR-143**	**HHT-CTL**	69.2	50.1–88.2	0.0336	66.7	43.0–85.4	90.5	69.6–98.8
	**HHT1-CTL**	74.2	49.9–98.5	0.0225	75.0	42.8–94.5	95.2	76.2–99.9
	**HHT2-CTL**	62.4	32.9–92.0	0.2876	55.6	21.2–86.3	90.5	69.6–98.8
	**HHT1-HHT2**	66.7	41.8–91.5	0.2009	33.3	7.5–70.1	100.0	73.5–100.0
**miR-150**	**HHT-CTL**	100.0	100.0–100.0	<0.0001	100.0	89.4–100.0	100.0	83.9–100.0
	**HHT1-CTL**	100.0	100.0–100.0	<0.0001	100.0	78.2–100.0	100.0	83.9–100.0
	**HHT2-CTL**	100.0	100.0–100.0	<0.0001	100.0	83.9–100.0	100.0	83.9–100.0
	**HHT1-HHT2**	60.0	39.7–80.3	0.3122	85.7	63.7–97.0	60.0	32.3–83.7
**miR-183**	**HHT-CTL**	54.4	38.7–70.1	0.5947	39.4	22.9–57.9	100.0	83.2–100.0
	**HHT1-CTL**	79.6	61.8–97.4	0.0057	75.0	42.8–94.5	85.0	62.1–96.8
	**HHT2-CTL**	73.8	61.8–97.4	0.0091	61.9	38.4–81.9	100.0	83.2–100.0
	**HHT1-HHT2**	81.4	66.2–96.5	0.0031	81.0	58.1–94.6	83.3	51.6–97.9
**miR-486**	**HHT-CTL**	98.8	96.7–100.0	<0.0001	100.0	90.3–100.0	90.5	69.6–98.8
	**HHT1-CTL**	99.5	98.1–100.0	<0.0001	100.0	81.5–100.0	95.2	76.2–99.9
	**HHT2-CTL**	98.2	94.9–100.0	<0.0001	100.0	81.5–100.0	90.5	69.6–98.8
	**HHT1-HHT2**	51.5	31.5–71.6	0.8743	61.1	35.7–82.7	61.1	35.7–82.7
**miR-654**	**HHT-CTL**	62.9	45.8–79.9	0.1435	57.1	37.2–75.5	100.0	81.5–100.0
	**HHT1-CTL**	62.8	38.7–86.9	0.2298	46.2	19.2–74.9	100.0	81.5–100.0
	**HHT2-CTL**	85.2	67.9–100.0	0.0006	80.0	51.9–95.7	100.0	81.5–100.0
	**HHT1-HHT2**	75.9	57.3–94.5	0.02	86.7	59.5–98.3	69.2	38.6–90.9

**Table 3:** Source of miRNAs. The miRNA expression was detected (+, yes; −, no) in macrophages, polarized into M1 and M2, endothelial cells (PAECs) and smooth muscle cells (PASMCs) in their respective cellular extracts (Cyto) and exosomes (Exo). MiR-103a was detected in all samples and used as internal reference.

	M1		M2		PAEC		PASMC	
	Cyto	Exo	Cyto	Exo	Cyto	Exo	Cyto	Exo
miR-9	+	+	+	+	−	−	−	−
miR-29c	+	+	+	+	+	+	+	+
miR-106b	+	+	+	+	+	+	+	+
miR-142	+	+	+	+	−	−	−	−
miR-143	+	−	+	−	+	−	+	+
miR-150	−	−	−	−	+	+	−	−
miR-183	+	+	+	+	−	−	−	−
miR-486	+	−	+	−	+	+	−	−
miR-654	−	−	−	−	+	−	+	+

## Data Availability

Raw miRNA-Seq data are available at the GEO repository (GSE179382).

## Supplementary Materials

The following are available online at https://www.mdpi.com/article/10.3390/ijms22179450/s1, Table S1: Complete list of differentially expressed exosome-transported miRNAs associated with HHT. Table S2: Biological processes affected in HHT according to the exosomal miRNA signature.
